# A Quick and Practical Approach to Secure a Chronic Fatigue Syndrome Diagnosis: The Novel Functional Limitation Index

**DOI:** 10.3390/jcm12227157

**Published:** 2023-11-17

**Authors:** Juan Antonio Corbalán, Gisela Feltes, Daniela Silva, Eduardo Gómez-Utrero, Iván J. Núñez-Gil

**Affiliations:** 1 Exercise Physiology Department, Vithas Arturo Soria Hospital, 28043 Madrid, Spain; corbalanja@vithasinternacional.com; 2 Cardiology Department, Vithas Arturo Soria Hospital, 28043 Madrid, Spain; feltesgg@vithas.es; 3 Faculty of Biomedical and Health Sciences, Universidad Europea de Madrid, Villaviciosa de Odón, 28670 Madrid, Spain; 4 Geriatric Department, Vithas International Arturo Soria Hospital, 28043 Madrid, Spain; silvasd@vithasinternacional.com; 5 Neurophysiology Department, Vithas International Arturo Soria Hospital, 28043 Madrid, Spain; eduardo.gomezutrero@salud.madrid.org; 6 Cardiology Department, Hospital Clínico San Carlos, 28040 Madrid, Spain

**Keywords:** Chronic Fatigue Syndrome, prognosis, diagnosis, ergospirometry, functional impairment, dyspnea

## Abstract

Chronic Fatigue Syndrome (CFS) is a serious, clinical, long-term condition with an unclear etiology and a difficult diagnosis. Our aim is to propose an objective physiological parameter (Functional Limitation Index, FLI) that describes the degree of functional impairment to support clinical suspicion. Materials and Methods: We consecutively included all CFS patients who consulted in the Exercise Physiology Department at our hospital, a dedicated referral unit for CFS, from 2009 to 2022. For comparison purposes, we included two control groups. Thus, three cohorts were included: the CFS group (patients with a previous definitive diagnosis), healthy voluntaries and a sportspeople/trained cohort (amateur athletes). All patients underwent a body composition test, spirometry, basal ECG in supine and standing positions and double peak effort ergospirometry with criteria of maximality. Results: The CFS+ group comprised 183 patients (85% female, mean age 46.2 years) and the CFS− included 161 cases (25.5% female, mean age 41.2 years); there were 93 patients in the healthy and 68 in the trained cohort. The CFS+ presented a lower functional class and scored worse in all of the performance parameters. The FLI was significantly higher in CFS+ (2.7 vs. 1.2; *p* < 0.001). The FLI displayed a good discrimination power (AUC = 0.94, *p* < 0.001), with a higher AUC than all of the other spirometric variables recorded. The best dichotomic overall FLI cutoff would be 1.66 with good specificity and sensitivity (S = 0.874, E = 0.864, Youden Index = 0.738). Conclusions: The Functional Limitation Index (FLI) could provide an easy and accurate diagnosis of this condition in both genders in a one-day assessment.

## 1. Introduction

Chronic Fatigue Syndrome (CFS), or myalgic encephalomyelitis, is a serious, long-term clinical condition that is still not fully understood by the medical community and has an unclear etiology [1,2]. It affects multiple body systems and presents a challenging diagnosis. The diagnosis is solely clinical, involving the exclusion of other pathologies due to the absence of supporting objective parameters, a clear pathophysiology, or a curative treatment [3]. The clinical suspicion that defines this condition encompasses five main symptoms: extreme fatigue, musculoskeletal pain, disrupted sleep, cognitive impairments and immune disorders. All of these symptoms are significant and have a profound impact on the patient’s quality of life. Due to its prevalence and disabling nature, numerous groups are researching CFS, although with significant variations in their approaches. This categorizes it among what are commonly referred to as rare diseases [4], and it has been suggested to be related to mitochondrial dysfunction.

Given that the primary symptom of the disease, although not the sole limitation experienced by the patient, is extreme fatigue or asthenia, the objective of our research is to propose a physiological parameter, termed the Functional Limitation Index (FLI), assessing peak effort capacity and recovery, based on years of dedicated experience.

This index describes the extent of functional impairment to support the clinical suspicion that defines this condition. The intention is not to replace other scoring systems or diagnostic questionnaires but rather to complement them.

## 2. Material and Methods

### 2.1. Study Group

We consecutively and prospectively enrolled all CFS patients who sought consultation at the Exercise Physiology Department at our hospital, a specialized referral unit for CFS, between 2009 and 2022. We did not employ formal exclusion criteria, and all eligible patients who underwent the test during this time period were included. For the purpose of comparison, we also included two control groups. Therefore, three cohorts, in each gender, were included in the current study, based on the following criteria:

1. CFS group: Patients already diagnosed with a definitive CFS by other clinical specialists, following the Fukuda diagnostic criteria [5] (Appendix A). These patients had limited symptoms, including those mentioned above and were referred to our clinic for that reason. Many of them required a study to assess disabilities. Patients with doubtful or incomplete CFS diagnoses were not included.
2. Healthy cohort: Healthy patients who wanted to collaborate, without any limiting disease or condition and who did not perform regular or scheduled physical activity. They were studied in the context of routine health checkups.
3. Sportspeople/trained cohort: Healthy athlete patients at an amateur level, understood as such, who trained weekly for more than 8–10 h of medium–high intensity (Mitchell Classification) [4].

### 2.2. Study Protocol and Procedures

All patients were initially subjected to a body composition test, using bioelectrical impedance analysis (BIA) technology. For the procedures in this study, we used a Schiller Cardiovit CS 200 ergospirometer, performing systematically: spirometry, basal ECG in supine and standing position and double peak effort ergospirometry (defined below), with criteria of maximality. Those were limited by extreme fatigue, other severe clinical limitation, or observer’s findings that could seriously compromise the patient’s safety.

In all cases, the patient was stimulated to achieve a QR (respiratory quotient) or an RER (respiratory exchange ratio) greater than 1.15, to provide us with an indication if their body was operating aerobically or anaerobically, as well as a cardiac reserve of less than 15% as the metabolic maximal criteria.

All patients included in the present study collaborated adequately and achieved the objectives set. All diagnostic procedures in this study were performed by a unique operator (JAC).

For the study of the patients, a protocol was used on a stationary bicycle specially adapted for patients with CFS that had the following stages:

1. Three minutes no-load pedaling warm-up.
2. Maximum test with progressive load of 15 w/m until the end of the test (maximum O_2_ consumption, VO_2_ max).
3. Rest of three minutes with rest on the bicycle or minimal pedaling, at the patient’s decision.
4. Supramaximal test started at the power equivalent to 50% of the maximum power reached in the test with a progression of 15 w every 10 s, until the exhaustion of the patient (supramaximal O_2_ consumption, VO_2_ smax).
5. Rest period, without pedaling, until the patient recovered completely.

During the test, blood pressure was automatically controlled using the brand’s own automatic sphygmomanometer, with continuous electrocardiogram tracing during the baseline, pre-effort, during exercise and in the recovery phase.

The tests were performed in the morning, mostly between 11 and 12 h. Taking into account that it is the best clinical phase for these patients who accumulate fatigue as the day goes by and are less functional in the afternoon. After the test and post-exercise recovery, the patient decided when to leave the laboratory. As in CFS patients, great efforts such as maximum ergospirometry generate a lot of fatigue and muscle pain; active rest was recommended the next day and analgesics were warranted according to the patient’s habit.

All patients signed the informed consent, and after a detailed explanation, all patients underwent the following procedures in this order:

1. Body composition study.

2. Spirometry.

3. Resting electrocardiogram.

4. Test with gas analysis

In all patients, a complete set of physiologic parameters was prospectively recorded (Appendix A). To sum up, age, gender, anthropometric data (weight, height, body mass index, % fat, muscle and water and muscle/fat ratio.), spirometric data (forced vital capacity -FVC-, expiratory volume in one second -FEV1-, FEV1/FVC, FEV at 25, 50 and 75%) and ergospirometric data (VO_2_ peak, VO_2_ in the anaerobic threshold -AT- (first threshold quotient ratio -QR = 1), VO_2_ entry into the lactic anaerobic phase (QR = 1.3), VO_2_ entry into the alactic anaerobic phase (QR = 1.10), measured in Supramaximal VO_2_ (SM VO_2_) at the end of recovery, O_2_ pulse (VO_2_/Heart ratio -HR) at AT and at peak effort, VO_2_/power-W- ratio, W peak and at each of the above thresholds, W/weight ratio, CO_2_ production, O_2_ and CO_2_ equivalents, VE peak, MVV (maximum voluntary ventilation, FEV1*40), ventilatory reserve and energy expenditure at rest, AT and peak effort) were analyzed according to the 9 Wasserman graphs and all the parameters were derived from them.

We developed an objective performance single value, the Functional Limitation Index (FLI), calculated as (VO_2_ peak/VO_2_ SM)/(VO_2_ SM/VO_2_ predicted).

All data were recorded in an anonymized electronic-dedicated database, and the study was conducted in compliance with the principles of the Declaration of Helsinki. All participants provided written informed consent to carry out all the mentioned procedures, the data of which are analyzed anonymously in this study.

### 2.3. Statistical Analysis

Clinical and physiologic data are presented with standard descriptive statistics: mean ± standard deviation (SD) or median ± interquartile range (IQR) when needed. Comparisons between groups were performed using the Pearson chi-squared for qualitative variables and the Student-t test or ANOVA tests when appropriate, for continuous variables. Discriminative capacity was assessed by the area under the ROC curve (AUC) and its 95% CI. Also, the Youden index was calculated to suggest an optimal FLI point.

A 2-tailed *p* < 0.05 was considered statistically significant. Statistical analyses and graphing were performed using the Office package 365 (Microsoft, Redmon, Washington, DC, USA), SPSS software v25 (IBM, Chicago, IL, USA) and R Core Team (2022), where R is a language and environment for statistical computing (R (version 4.2.2) Foundation for Statistical Computing, Vienna, Austria. URL https://www.R-project.org/ (accessed on 8 September 2023)).

## 3. Results

**Figure 1:**
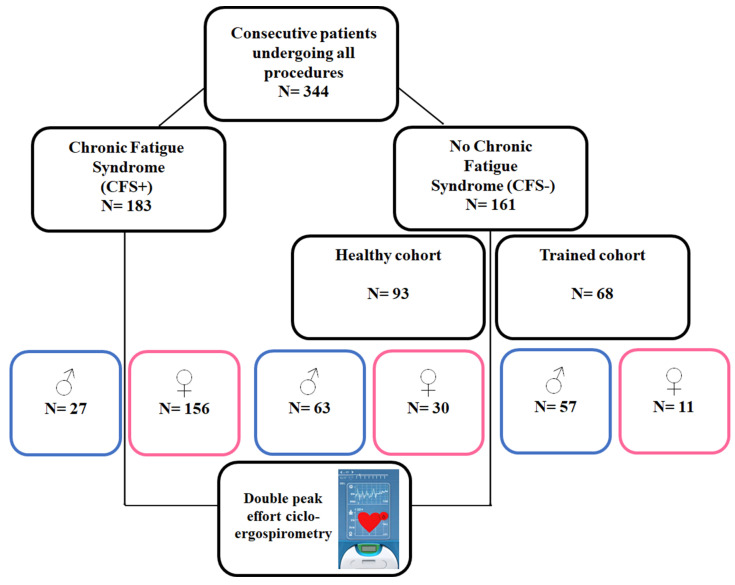
Study patient flow-chart.

Finally, we included 344 patients in the present analysis, Figure 1. The CFS+ group comprised 183 patients (85% female, mean age 46.2 years old) and the CFS− included 161 cases (25.5% female, mean age 41.2 years). For the CFS−, 93 patients were in the healthy and 68 in the trained cohort. Table 1 depicts the main physiologic parameters measured regarding the presence of CFS or not.

The CFS+ presented a lower functional class and they scored worse in all the performance parameters. The FLI was significantly higher in the CFS+ (2.7 vs. 1.2; *p* < 0.001).

Table 2 provides the findings regarding the cohort and gender. The previously shown trend is maintained regarding the training status, with incremental performance in the trained group.

**Figure 2:**
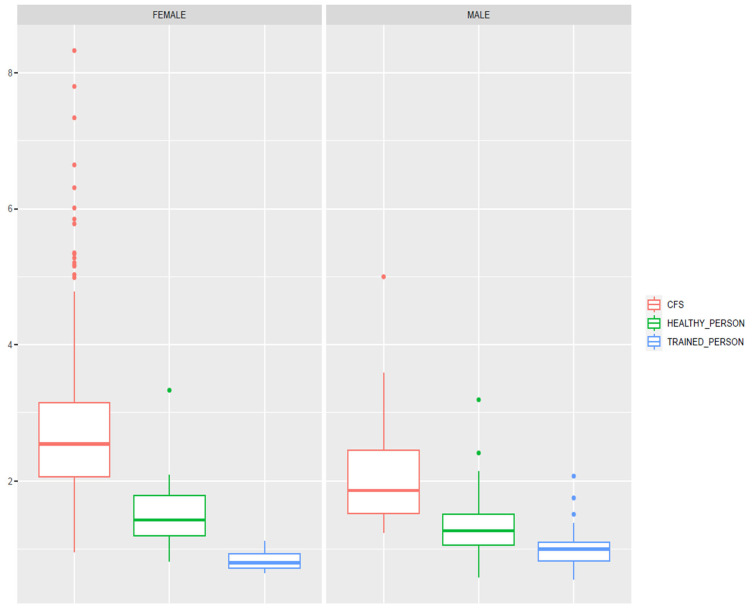
Boxplot graphs, displaying FLI values regarding the comparison groups and genders. Lower FLI values correspond with higher health status and training level in both genders. FLI: Functional Limitation Index. CFS: Chronic Fatigue Syndrome.

Figure 2 depicts the boxplot regarding FLI values, stratified by gender and CFS status. FLI values are higher in female and CFS patients. Trained people presented lower FLI values compared with the other two groups. The main Z-scores for FLI are shown in Table 3.

**Figure 3:**
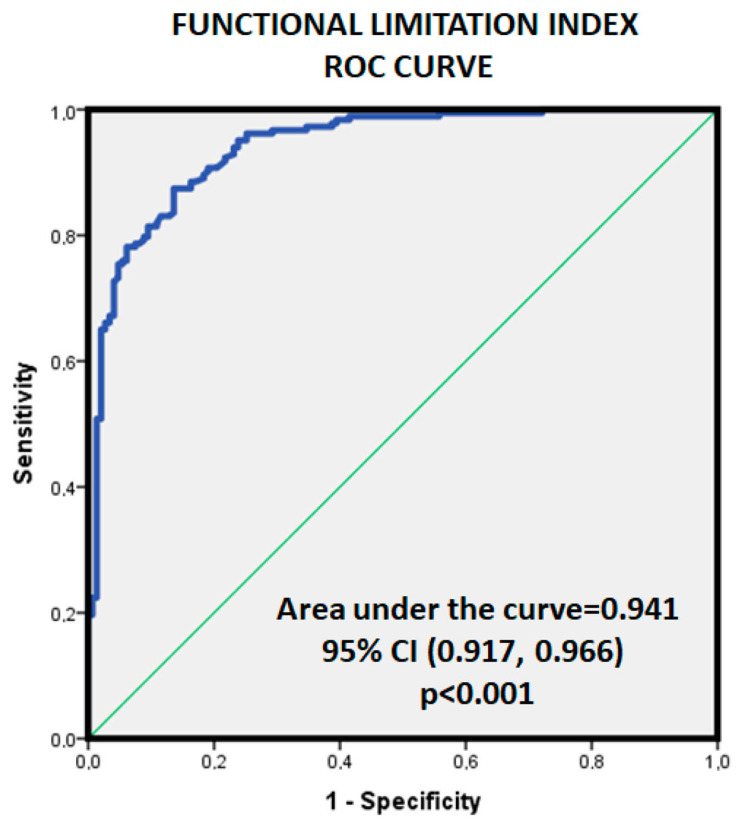
Area under the curve for the Functional Limitation Index regarding the existence or not of pathology (CFS+ vs. CFS−).

Figure 3 displays the ROC curve, regarding CFS or not for FLI with a good discrimination power (AUC = 0.94, *p* < 0.001). After calculating the Youden index, the best dichotomic overall FLI cutoff would be 1.66 with good specificity and sensitivity (S = 0.874, E = 0.864, Youden Index = 0.738).

Also, FLI was associated with the best discriminative power, higher AUC of all variables recorded (see Table 1) in this study, followed by BR (<35%) (AUC = 0.87, 95%CI = 0.79–0.94; *p* < 0.001) and VO_2_m/sm (AUC = 0.84, 95%CI = 0.75–0.94; *p* < 0.001).

Since the women’s FLI values were higher in all groups, we repeated the assessment stratified by gender. Considering only females, the AUC for FLI was 0.92 (*p* < 0.001, 95%CI = 0.88–0.97), and the dichotomic FLI cutoff was 1.94 (S = 0.814, E = 0.925, Youden Index = 0.739). For male FLI, the AUC for FLI was 0.91 (*p* < 0.001, 95%CI = 0.86–0.96), and the gender-adjusted dichotomic FLI cutoff would be 1.41 (S = 0.96, E = 0.813, Youden Index = 0.776).

## 4. Discussion

### 4.1. General Considerations

This study includes, to the best of our knowledge, one of the largest series reported with CFS and depicts their complete physiological assessment from the point of view of the physiology of the exercise. Also, we provide a full comparison with a two-level control cohort (CFS−) comprising regular non-trained but healthy people and a nonprofessionally trained cohort and provide a single robust parameter useful to support a CFS diagnosis.

The main findings are as follows:

The VO_2_ peak is clearly decreased in patients with CFS, more in women than in men and below 80% of their predicted VO_2_ as compared to the normal population and much more if compared to the sports population. Most of them would fit into a 2–3 Functional Class Group of the NYHA.

The W peak is decreased to a lesser degree than the VO2, but in figures below normality.

A VO_2_ SM ratio against the theoretical below 60% is found in patients with CFS.

A high VO_2_ max/VO_2_ (SM/Teo) ratio is observed in the CFR+ cohort.

The FLI is higher in patients with CFS (>1.5) compared to normal patients (between 1 and 1.5) and, of course, compared to athletes (tendency of <1). These values are influenced by athletes with very little physical requirements and under training who behave like normal people and by very sedentary people who can border on pathological values.

This parameter (FLI) presents a pretty good discriminative ability for CFS diagnosis, in both genders.

The O_2_ pulse is clearly below that predicted (<80%), which does not occur in normal patients or athletes (inotropic function).

The AT appears below 40–50% of the normal VO2 peak and above 50–60% of that which increases with regular or good patient preparation. In long-distance athletes, it can appear beyond 80–85% (suitability criteria to withstand a greater aerobic load).

Consequently, these findings could comprise major criteria for supporting the diagnosis of CFS. Moreover, the physiological assessment displayed here for CFS would be able to discard simulators. Thus, the FLI arises as a very accurate and robust parameter for an easier and quicker CFS diagnosis. Henceforward, a correct CFS diagnosis is of paramount importance not only because of reimbursement policies but mainly in order to orientate the diagnostic or therapeutic management of a complex and limited patient in a multimodality setting.

Similar results to those presented above can be seen in the scientific literature [1,2]. CFS has been classified as a rare disease, with a difficult diagnosis and no curative treatment. An unknown etiology [3] and pathophysiology [4,5,6,7,8] lead us to a complex multisystemic clinical problem, which advises close collaboration among multiple specialties, such as cardiology, internal medicine, neurology, and sports medicine, among others.

### 4.2. Cardiopulmonary Exercise Testing in CFS

The main symptom, although not the only limiting one, is the loss of functional capacity, which is not exclusive to CFS. Therefore, the use of the Cardiopulmonary Exercise Test Methodology (CPET) is essential for a proper diagnosis and should be applied to all patients with clinical suspicion [9,10]. The choice of using a bicycle or a treadmill, maximal or submaximal tests, or one- or two-day tests [11] remains to be determined. Many recommend using a bicycle due to the instability and lack of ability to engage in physical activity for many patients. The disadvantage is that a significant portion of patients, due to their lack of familiarity with pedaling, may not reach their maximum oxygen consumption (VO_2_ max). It is estimated that there could be an underestimation of approximately 10–15% compared to the treadmill test, depending on the patients’ adaptation to the treadmill. On the other hand, some researchers have suggested the convenience of conducting the Cardiopulmonary Exercise Test Methodology tests over two days, which may pose logistical and emotional difficulties, although this has been debated by others [12], and it is left to the discretion of the responsible physician. In our experience, a one-day test with a double peak of maximum effort could be as useful as the two-day test, possibly with a better cost-benefit ratio and less demand for the patient and the healthcare system.

### 4.3. CFS Pathophysiology

From a pathophysiological perspective, in CFS, there appears to be an underlying mitochondrial dysfunction [13]. In these patients, an increase in intramitochondrial pyruvic acid has been described, which, according to the law of mass action [14], would hinder the penetration of cytoplasmic pyruvic acid into the mitochondria formed during the degradation of glucose under increasing high-intensity exertion. This would lead to a consequent increase in lactic acid, a precursor of fatigue. Lactic acid itself does not limit ergogenic capacity; it behaves as an emergency metabolic pathway to maintain our functional capacity at the cost of increasing fatigue due to acidosis, initially locally and subsequently generalized, under anaerobic conditions where it collaborates with other substrates in the incipient and partial regeneration of glycogen [15], whose end metabolite, pyruvic acid, cannot enter the aforementioned organelle.

Supporting this theory, without disregarding other affected organs, asthenia, memory loss, impaired neurovegetative function and susceptibility to infections could be the result of partial organ failure in those tissues with higher mitochondrial density (skeletal and cardiac muscle, nervous and immune systems, etc.) [8,16]. Additionally, the activity of free radicals could contribute to a pro-inflammatory state that further facilitates the entire process [17].

The decrease in functional capacity at rest and, of course, during exercise, leading patients to be classified in NYHA functional classes two to four, is a constant in these patients [18]. Similarly, moderate to severe impairment in cognitive assessment tests could be the result of the profound involvement of skeletal and cardiac muscle, the dysfunction of the central and peripheral nervous system and the deterioration of the immune system.

The impairment in VO_2_, the generalized post-exertional disorder and the accompanying malaise experienced by patients in the following days could be related to the immediate and sustained clinical symptoms of these patients.

### 4.4. CFS and FLI Rationale

The objective of this study, the FLI, is to compare the response and behavior of the patient between the first peak of VO_2_ in a conventional test using the protocol commonly used for these patients and a second, shorter but more intense peak, after three minutes of recovery. The rationale behind this study stems from the idea that the ability to repeat a maximal effort with minimal recovery time depends on the level of conditioning of all the organs and systems involved in oxygen consumption (VO_2_). In sports medicine, a series training system is used to train speed endurance (expression of maximal effort). When an athlete runs at 80–85% of their maximum capacity over a set distance of, for example, 100 to 300 m, the possibility of equaling the time in a second repetition of the effort decreases with the number of repetitions. However, the second repetition can be equal or very close to the first, especially in shorter distances. In essence, it is possible to achieve the same peak VO_2_ in the second effort as in the first if the athlete’s preparation is appropriate. Conversely, the second test will progressively be worse with a lower physical condition of the participant, regardless of the cause, whether it is physical deconditioning or illness.

Thus, in an athlete, this ratio (VO_2_ max/VO_2_ SM) could be one or even less than one, as the improvement stimulus could be a factor to consider in the second effort. A second possibility we see in the laboratory is that the patient presents with asthenic symptoms of a different etiology than CFS. In this case, we would expect the second effort to yield a VO_2_ SM clearly lower, albeit not much, than the first effort. In this scenario, the aforementioned ratio should always be greater than one. This also includes individuals without any known pathology or with a metabolic profile different from CFS. This led us to explore the responses of a group of healthy individuals as controls in the present study (see Z-scores).

The third possibility would be the case of patients with CFS. In these patients, the ratio between the first and second efforts is much higher due to the degree of mitochondrial and metabolic impairment in their response. Therefore, the numerator mentioned above in the form of a ratio would be significantly higher, on the order of >1.5, as observed in the analysis of our results. In addition to the objective test data, these individuals exhibit a symptomatic profile with profound physical fatigue and mental fog. This symptomatology persists depending on the degree of patient impairment and varies in the following days. This ratio would serve as the numerator of the fraction that defines the FLI (see Section 2).

To complete the FLI, as the denominator, we used the ratio between the second peak and the theoretical maximum VO_2_ corresponding to the patient’s gender and age (VO_2_ SM/predicted theoretical VO_2_ max; i.e., the ratio of the fatigue test to the theoretical value). The analysis of this ratio also provides three possibilities. In athletes, the chances of achieving a ratio of one are maximized, meaning it would be 100% or even higher in percentage. Despite the first effort, well-trained individuals reach or come very close to the values of the first peak. In the case of healthy individuals, the ratio may slightly decrease below one, with a percentage of the theoretical value approaching 100% or slightly below (70–90% according to the results), depending on their level of physical fitness resulting from an active lifestyle. The third case is for patients with CFS, who always have a significant reduction in relation to the theoretical VO_2_, generally below 60% as observed in the analysis of our findings.

Under normal conditions, therefore, the fraction that makes up the FLI would tend to be 1 or 100%, or slightly lower. Athletes would have values below one, with a greater difference depending on their level of training, while patients with CFS would clearly have values above one, also varying according to the degree of impairment.

Finally, FLI offers a quick approach to obtaining objective data on one of the main limitations depicted in CFS which could complement some of the main SFC diagnostic criteria. One commonly used diagnostic criterion is the “Fukuda Criteria” [5] which was developed by the Centers for Disease Control and Prevention (CDC) and is the set accepted in the present study as selection criteria (Appendix A). The Fukuda Criteria include, as a main complaint, a significant decrease in the ability to engage in pre-illness levels of occupational, educational, social or personal activities, along with fatigue that lasts for at least 6 months and is of new or definite onset. Also, fatigue should not be alleviated by rest, as can be pointed out by FLI. Additionally, there are other diagnostic criteria and scales that have been used in research and clinical practice to diagnose CFS, such as the Canadian Consensus Criteria and the International Consensus Criteria [19,20]. These criteria vary in their requirements and are used in combination with patient-reported symptom scales [21]. Since up to 90% of people with CFS [21] are estimated to be undiagnosed, all efforts to ease it are warranted [22,23].

## 5. Limitations

Most of the participants in this study were relatively young adults, mostly less than 50 years old. Therefore, the generalization of our physiological findings to other age segments should be performed cautiously. Additionally, we did not have precise data on the race, with the majority being Caucasian. Therefore, any extrapolation to other racial groups should be approached with caution.

As usual, since the design is observational and cross-sectional, we could not discard some potential biases, and we could not inform on the impact of training in future CFS evaluations or prognosis. Potential differences in the epidemiological profile of the cohorts studied here could derive in some selection biases also.

Anyway, we think that the careful and complete physiological evaluation of the patients of this protocol provides relevant information for the real-life practice of the clinician involved in the care of CFS patients, making the FLI determination an easy and robust parameter to support a CFS diagnosis. The present study was performed including patients with established CFS diagnosis. Regarding earlier stages of development further studies are needed to define the usefulness of FLI.

## 6. Conclusions

The exercise physiology parameters determined with ergospirometry are greatly altered in patients with CFS. A unique and simple parameter, such as the Functional Limitation Index (FLI), could provide an easy and accurate diagnosis of this condition in both genders in one-day assessment.

Further studies are needed to determine if exercise parameters could provide an adequate estimation of future outcomes or therapeutic responses.

## Figures and Tables

**Table 1:** Findings regarding the presence or not of chronic fatigue syndrome diagnosis (CFS+ vs. CFS−).

Variable	CFS+	CFS−	*p*-Value
Gender (Female)	85.2%	25.5%	**<0.001**
Age	46.2 (9.3)	41.2 (13.8)	**<0.001**
Weight	66.3 (12.9)	77.3 (14.3)	**<0.001**
VO_2_m	20.4 (6.3)	38.0 (9.6)	**<0.001**
VO_2_m/t	76.8 (16.7)	114.8 (24.1)	0.093
Wmax	118.2 (33.4)	220.86 (58.9)	**<0.001**
W m/t	87.4 (19.0)	122.9 (25.5)	**<0.001**
W/K	87.4 (19.0)	3.04 (0.7)	**<0.001**
FC M/T	84.7 (9.8)	93.0 (10.7)	**<0.001**
FC AT	120.5 (20.7)	137.5 (18.1)	**<0.001**
VO_2_/W	11.38 (1.9)	13.0 (1.7)	**<0.001**
VO_2_m/sm	140.4 (31.2)	114.3 (13.2)	**<0.001**
VO_2_sm/t	57.1 (15.0)	100.8 (25.2)	**<0.001**
FLI	2.7 (1.26)	1.2 (0.4)	**<0.001**
VO_2_sm/m	0.7 (0.1)	0.9 (0.0)	**<0.001**
VE	46.1 (14.3)	90.1 (25.1)	**<0.001**
EQO2	33.5 (5.5)	31.5 (4.1)	**<0.001**
VE/VCO_2_	27.2 (5.3)	24.8 (3.6)	**<0.001**
Pulse _O2_	9.0 (2.5)	17.3 (4.9)	**<0.001**
VEF1	2.65 (0.7)	3.7 (0.7)	**<0.001**
MVV	70.5 (54.7)	147.4 (30.1)	**<0.001**
V.ESTIM	51.5 (12.1)	85.4 (24.5)	**<0.001**
BR < 35%	55.2 (12.3)	37.9 (13.3)	**<0.001**

Numeric variables are depicted as MEAN (SD). Acronyms provided in methods and in the abbreviature list. The values highlighted in bold indicate statistically significant differences.

**Table 2:** Parameters regarding study group and gender.

Variable	CFS+ Female	CFS+ Male	Healthy Female	Healthy Male	Trained Female	Trained Male	*p*-Value *
Age	46.8 (8.9)	42.8 (11.3)	43.6 (9.4)	46.1 (13.7)	32.7 (10.3)	36.1 (14.2)	**<0.001**
Weight	64.6 (11.9)	77.2 (14.4)	61.3 (12.9)	81.2 (12.0)	59.4 (5.9)	77.2 (11.4)	**<0.001**
VO_2_m	19.3 (5.1)	26.7 (8.5)	28.6 (6.3)	33.9 (5.7)	42.3 (5.9)	46.6 (7.4)	**<0.001**
VO_2_m/t	77.0 (16.7)	75.4 (16.5)	104.6 (19.2)	105.1 (17.1)	148.0 (28.5)	124.54 (23.0)	0.350
Wmax	109.5 (24.3)	168.1 (35.5)	147.9 (26.6)	213.3 (38.5)	200.5 (25.9)	271.5 (38.6)	**<0.001**
W m/t	87.8 (19.2)	85.1 (18.6)	118.9 (19.6)	110.3 (22.7)	155.5 (20.2)	132.6 (24.8)	**<0.001**
W/K	1.73 (0.4)	2.27 (0.7)	2.45 (0.5)	2.67 (0.4)	3.39 (0.4)	3.6 (0.6)	**<0.001**
FC M/T	83.9 (9,7)	89.1 (9.4)	94.5 (14.2)	92.7 (7.3)	92.4 (4.5)	92.8 (12.6)	**<0.001**
FC AT	121.5 (21.6)	114.8 (14.8)	129.0 (16.8)	135.4 (17.9)	142.6 (33.3)	142.3 (13.9)	**<0.001**
VO_2_sm	13.9 (4.3)	22.1 (7.3)	23.6 (6.3)	29.9 (5.9)	38.5 (5.3)	42.8 (7.6)	**<0.001**
VO_2_m/sm	143.5 (31.9)	123.2 (19.1)	123.1 (12.7)	115.0 (14.9)	109.0 (6.2)	109.3 (9.3)	**<0.001**
VO_2_sm/t	56.1 (14.9)	62.6 (14.1)	85.9 (19.0)	91.4 (19.1)	135.2 (23.1)	112.9 (22.7)	**<0.001**
FLI	2.84 (1.3)	2.12 (0.8)	1.52 (0.4)	1.33 (0.4)	0.83 (0.1)	1.01 (0.27)	**<0.001**
VO2sm/m	0.72 (0.1)	0.82 (0.1)	0.82 (0.1)	0.88 (0.1)	0.91 (0.0)	0.92 (0.1)	**<0.001**
VE	42.8 (11.9)	65.3 (12.3)	58.9 (12.5)	87.9 (16.0)	84.1 (13.9)	110.2 (21.1)	**<0.001**
EQO2	33.8 (5.7)	31.7 (3.8)	33.6 (4.2)	31.4 (4.0)	32.9 (2.6)	30.4 (3.9)	**<0.001**
VE/VCO_2_	27.9 (5.2)	23.7 (4.1)	26.0 (3.8)	24.7 (2.9)	24.3 (2.9)	24.3 (4.1)	**<0.001**
Pulse O_2_	8.4 (1.8)	12.8 (2.6)	10.3 (1.9)	17.4 (3.3)	14.5 (2.1)	21.5 (2.8)	**<0.001**
VEF1	2.51 (0.5)	3.89 (0.6)	2.77 (0.4)	3.82 (0.6)	3.01 (0.3)	4.13 (0.6)	**<0.001**
MVV	63.6 (51.3)	147.7 (23.6)	110.8 (16.1)	152.8 (23.0)	120.4 (13.7)	165.3 (24.0)	**<0.001**
V.ESTIM	48.4 (8.7)	69.5 (12.8)	62.2 (9.6)	85.8 (13.8)	39.3 (12.3)	106.7 (13.9)	**<0.001**
BR < 35%	55.2 (12.6)	55.2 (10.5)	46.3 (12.2)	41.2 (11.0)	29.7 (11.4)	32.6 (13.2)	**<0.001**

* ANOVA between groups. Acronyms provided in methods and in the abbreviature list. The values highlighted in bold indicate statistically significant differences.

**Table 3:** Functional limitation index (FLI) Z-Score table.

Study Group	FLI Mean Z-Score (SD)	*p*-Value
Gender (all)		**<0.001**
− Female − Male	0.38 (1.07) −0.56 (0.52)	
Health status		**<0.001**
− CFS+ − CFS−	0.55 (1.02) −0.68 (0.35)	
CFS + Gender		**0.006**
− Female − Male	0.63 (1.05) 0.05 (0.67)	
CFS – Gender		0.072
− Female − Male	0.43 (0.43) 0.32 (0.32)	
Training level		<0.001 *
− CFS+ − Healthy cohort − Trained cohort	0.55 (1.02) −0.54 (0.37) −0.88 (0.21)	

* ANOVA comparison. The values highlighted in bold indicate statistically significant differences.

## Data Availability

Our data are available upon reasonable request.

## Abbreviations

BR < 35%	Ventilatory reserve (maximum ventilation vs. voluntary ventilation VMV)
EQO2	O_2_ equivalent
FEV1	Forced Expiratory volume in one second (spirometry)
FLI	Functional limitation index
FVC	Forced Vital Capacity
HR AT	Heart rate at the anaerobic threshold (VT1)
HR M/T	Maximum heart rate achieved with respect to the theoretical maximum
Pulse O_2_	O_2_ pulse vs. heart rate (inotropic function)
MVV	Maximum voluntary ventilation (FEV1*40, convention)
VE	Ventilation
V.ESTIM	Estimated ventilation.
VO_2_m	Peak (maximum) O_2_ consumption
VO_2_m/t	Peak O_2_ consumption reached compared with the theoretical
VO_2_m/sm	Relationship between maximum O_2_ consumption and supramaximal (second peak)
VO_2_sm/t	Supramaximal consumption (2 peak) vs. theoretical
VO_2_sm/m	Supramaximal O_2_ consumption (second peak) vs. máximum (first peak)
VE/VCO_2_ (EQ. CO_2_)	CO_2_ equivalent
VO_2_/W	Relationship between O_2_ consumption and power
Wmax	Peak (Maximum) power
W m/t	Peak (Maximum) power compared with the theoretical
W/K	Power to weight ratio

## Appendix A

### Appendix A.1. Data (Prospectively) Recorded by Protocol in the Present Study

− Age, gender.
− Anthropometric Data: Weight, height, BMI, % fat, muscle and water, Fat/Muscle Ratio, BMI-Corrected.
− Spirometric Data: FVC, FEV1, FEV1/FVC, FEV at 25.50 and 75%, classified later in the Muller graph.
− Ergospirometric data: They were analyzed according to the 9 Wassermman graphs and all the parameters derived from them.
  - VO_2_ peak, VO_2_ in AT (first threshold QR = 1), VO_2_ entry into the lactic anaerobic phase (QR = 1.3), VO2 entry into the alactic anaerobic phase (QR = 1.10), measured in Supramaximal VO_2_ (SM VO_2_) at the end of recovery.
  - VO_2_ peak/VO_2_ SM
  - FLI. Functional Limitation Index (VO_2_ peak/VO_2_ SM)/(VO_2_ SM/VO_2_ predicted)
  - O_2_ pulse (VO_2_/HR) at AT and at peak effort.
  - VO_2_/W ratio
  - W peak and at each of the above thresholds
  - Power/weight ratio
  - CO_2_ production
  - O_2_ and CO_2_ equivalents
  - PTE of O_2_ and CO_2_
  - VE peak
  - MVV (FEV1*40)
  - Ventilatory Reserve (BR, Breath Reserve) (VMV − VE peak)/VMV, in %
  - Energy expenditure at rest, AT and peak effort

### Appendix A.2. Fukuda et al., CFS Diagnostic Criteria (1994) [5]

1. Persistent fatigue (at least six months) or intermittent, unexplained, recurring or with a defined onset, not resulting from recent efforts; does not improve with rest; leads to a noticeable reduction in the patient’s previous usual activity.
2. Exclusion of other diseases that can cause chronic fatigue.

Concurrently, four or more signs or symptoms must be present, all of them persistent for six months or more and occurring after the onset of fatigue.

1. Recent concentration or memory impairments.
2. Odynophagia.
3. Painful cervical or axillary lymph nodes.
4. Myalgias.
5. Polyarthralgias without signs of inflammation.
6. Recently onset headaches or headaches with different characteristics from the usual ones.
7. Non-refreshing sleep.
8. Post-exertional malaise lasting more than 24 h.
